# Prognostic factors for disease-specific survival in 108 patients with Hürthle cell thyroid carcinoma: a single-institution experience

**DOI:** 10.1186/1471-2407-14-777

**Published:** 2014-10-23

**Authors:** Rok Petric, Barbara Gazic, Nikola Besic

**Affiliations:** Department of Surgery, Institute of Oncology Ljubljana, Ljubljana, Slovenia; Department of Pathology, Institute of Oncology Ljubljana, Ljubljana, Slovenia

**Keywords:** Hürthle cell thyroid carcinoma, Surgery, Survival, Pathology

## Abstract

**Background:**

Hürthle cell thyroid carcinoma (HCTC) is a rare disease. It is believed that it is more aggressive than follicular thyroid carcinoma. The aim of our study was to identify factors associated with disease-specific and disease-free survival.

**Methods:**

Altogether, 108 patients with HCTC (26 male, 82 female; median age 62 years; range 19–87 years) treated at our Institute from 1972 to 2011 were included in the present retrospective study. Data on age, clinical and histopathological factors, tumor stage, recurrence, disease-free and disease-specific survival were collected. Univariate analysis was used to identify factors associated with disease-specific survival. Cox’s multivariate regression model was used to identify independent prognostic factors for disease-specific survival.

**Results:**

The follow-up period was 1 to 337 (median 105) months. Of 108 patients, 12 (11%) had distant and 8 (7%) had locoregional metastases before primary treatment. Recurrence was diagnosed in 26 cases (24%): locoregional, distant, and both locoregional and distant in 12, 11, and 3 cases, respectively. The 5-year, 10-year, and 20-year disease-specific survival were 96%, 88%, and 67%, respectively. Independent prognostic factors for disease-specific survival were: age of patients at diagnosis, distant metastases and residual tumor after surgery.

**Conclusion:**

Long disease-specific survival was found in patients with HCTC younger than 45 years of age without distant metastases and without residual tumor after surgery.

## Background

Hürthle cell thyroid carcinoma (HCTC) is a rare type of thyroid carcinoma [1] which accounts for around 3% of all thyroid malignancies [2]. Only about 400 patients with HCTC were reported from 1935 to 2004 [1]. According to the World Health Organization classification [3], HCTC is considered an oxyphilic variant of follicular thyroid cancer (FTC), but genomic dissection of HCTC revealed a unique class of thyroid malignancy distinct from papillary and follicular thyroid cancer [4].

There are only few population-based studies [2, 5–7] and many reports from single-institution studies of patients with HCTC [8–24]. Older studies reported poor survival of patients with HCTC [8, 9, 11–13], but patients were not treated in accordance with the current standard of care. Nagar et al. reported that survival in HCTC has improved dramatically over time in the United States and that survival rates for HCTC and FTC are currently the same [7]. Similarly, Bhattacharyya reported that overall survival for HCTC was similar to that of comparably staged FTC [2]. Unfortunately, they did not report on the treatment of patients, and they did not explain why survival of patients with HCTC has improved.

At our Institute, patients with HCTC have been treated over the last 40 years on the basis of the same principle, which is now standard treatment and is recommended by the ATA guidelines [25]. Our aim was to report a single-institution experience with a large number of patients with a long follow-up period. Another aim of the study was to determine which factors are predictive of recurrence and disease-specific survival in HCTC.

## Methods

### Patients

A total of 108 patients with HCTC (82 women, 26 men; age 19 to 87 years, median age 62 years, mean age 58 years) were treated at our Institute between 1975 and 2007 and were included in our retrospective study. The follow-up time was 1–337 (median 105, mean 117) months.

The Medical Ethics Committee of the Republic Slovenia and the Protocol Review Board and Ethics Committee of the Institute of Oncology Ljubljana reviewed and approved the study, which was performed in accordance with the ethical standards laid down in an appropriate version of the 1964 Declaration of Helsinki.

All histological slides were examined by the pathologist (B.G.) experienced in thyroid pathomorphology. A diagnosis of HCTC was made based on histological criteria defined by LiVolsi and Rosai [26, 27]. Only lesions demonstrating more than 75% of follicular cells with oncocytic characteristics were included in the study group. The identification of a cell as an oncocyte was based on the presence of acidophilic, granular cytoplasm and hyperchromatic or vesicular nuclei with large nucleolus. The diagnosis of malignancy was based on histological evidence of transcapsular and/or vascular invasion, extrathyroidal local tissue invasion by primary tumor [26, 27], or presence of nodal or distant metastasis. All patients with Hürthle cell neoplasms with cells containing typical nuclear features of papillary carcinoma were excluded from our present study and were the subject of one of our previous studies [28].

A chart review was performed, and data on patients’ age, clinical and histopathological factors, tumor stage, treatment, recurrence, disease-free and disease-specific survival were collected. Clinical and pathomorphological characteristics are given in Table 1.

**Table 1:** **Patients’ and tumor characteristics and univariate statistical analysis of disease-specific survival**

Factor	Subgroup	Patients number (%)	Dead of disease (N = 20)	Alive or dead of other causes (N = 88)	p-value
**Gender**	Female	82 (76)	12	70	0.08
	Male	26 (24)	8	18	
**Age (years)**	44 or less	23 (21)	0	23	0.006
	45 or more	85 (79)	20	65	
**Tumor diameter (cm)**	0-4	56 (52)	2	54	0.001
	4.01 and more	52 (48)	18	34	
**pT tumor stage**	pTx	1 (1)	1	0	0.001
	pT1	9 (8)	0	9	
	pT2	35 (32)	1	34	
	pT3	47 (43)	9	38	
	pT4	16 (15)	9	7	
**N stage**	N0	100 (93)	16	84	0.04
	N1 (N1a + N1b)	8 (7)	4	4	
	N1a	3			
	N1b	5			
**M stage**	M0	96 (89)	11	85	0.001
	M1	12 (11)	9	3	
**Tumor differentiation**	Well	66 (61)	8	58	0.03
	Moderate or poor	36 + 6 (33 + 6)	12	30	
**Vascular invasion**	Minimal or suspected	66 (61)	10	56	0.26
	Extensively	42 (39)	10	32	
**Capsular invasion**	No or minimal	56 (52)	8	48	0.24
	Transcapsular	52 (48)	12	40	
**Thyroid surgical procedure**	Total or near-total thyroidectomy	77 (71)	8	69	0.001
	Lobectomy or less	31 (29)	12	19	
**Residual tumor after surgery**	R0 – without residual tumor	80 (74)	7	73	0.001
	R1 – microscopic residual tumor	19 (17)	10	9	
	R2– macroscopic residual tumor	9 (8)	3	6	
**Neck dissection**	No	99 (92)	15	84	0.01
	Yes	9 (8)	5	4	
**Radioiodine ablation after surgery**	No	21 (20)	5	16	0.53
	Yes	87 (80)	15	72	
**Therapy with radioiodine**	No	77 (71)	3	29	0.17
	Yes	31 (29)	17	59	
**External beam radiotherapy**	No	85 (79)	11	74	0.004
	Yes – neck and mediastinum	23 (21)	9	14	
**External beam radioterapy**	No	81 (75)	6	75	0.001
	Yes – any site	27 (25)	14	13	
**Chemotherapy**	No	92 (85)	11	81	0.001
	Yes	16 (15)	9	7	
**Recurrence**	No	70 (65)	0	70	-
	Yes - distant	11 (10)	4	7	
	Yes – locoregional	12 (11)	5	7	
	Yes – locoregional and distant	3 (3)	2	1	
	Initially distant metastases	12 (11)	9	3	
**Outcome**	Alive without disease	52 (48)	0	52	-
	Alive with disease	16 (15)	0	16	
	Dead of disease	20 (19)	20	0	
	Dead of other causes	15 (14)	0	15	
	Lost to follow-up	5 (5)	0	5	

Patients were categorized into two groups: less than 45 years of age and 45 years or older. Tumor size, presence of regional and/or distant metastases, as well as residual tumor after surgery were assessed by the TNM clinical classification system according to the UICC criteria from 2009 [29]. In addition to chest X-ray, the diagnostic work-up also included ultrasound (US) of the thyroid gland and determination of serum thyroglobulin (Tg) concentration. Additional diagnostic work-up (computed tomography [CT], magnetic resonance imaging [MRI], bone scintigraphy and/or PET-CT) was performed whenever medical history or physical examination indicated distant metastases. After thyroid surgery, additional diagnostic work-up was performed if radioactive iodine (RAI) uptake was detected outside the thyroid bed, or if the thyroglobulin (Tg) level was greater than 1 ng/mL.

Follow-up was performed at our Institute at least once a year. It consisted of a medical history, physical examination and determination of serum Tg concentration. Criteria for disease-free survival were: Tg levels of less than 1 ng/mL, negative whole-body RAI scans, and exclusion of cervical lymph node metastases detected by US [25, 30]. Whenever Tg concentration was elevated or clinical symptoms of possible recurrence were present, imaging (X-ray, US, CT, MR, bone scintigraphy, PET-CT and/or RAI scintigraphy) was performed in order to determine the site and extent of suspected recurrence.

### Treatment

Our patients were treated by different surgeons. Therefore, surgical treatment of primary tumor, locoregional recurrences, and distant metastases was not uniform; neither was the proportion of patients treated by RAI ablation of thyroid remnant, external beam radiotherapy (EBRT), chemotherapy, and RAI therapy.

Surgery is the mainstay of the treatment of HCTC. Among surgically-treated patients, 79% had primary surgery at the Institute of Oncology and 21% elsewhere (some of them had either biopsy alone or some kind of inadequate surgery). Completion thyroidectomy was performed in 20 patients. All other specific therapies (surgery, RAI, EBRT and/or chemotherapy) as well as follow-up were performed in all patients at the Institute of Oncology.

Total or near-total thyroidectomy is considered an adequate surgical procedure for HCTC. Our treatment approach after an inadequate surgical procedure is generally completion thyroidectomy. However, completion thyroidectomy was not performed if there were technical problems (e.g. recurrent nerve injury or hypoparathyroidism or severe fibrosis), or if the patient refused another surgical procedure or preferred treatment with RAI. Initial treatment in 13 patients with locally advanced tumor was neoadjuvant chemotherapy, while one patient received concomitant EBRT with a tumor dose of 36 Gy. Tumor size decreased in all these patients, and in 43%, the largest tumor size decreased by more than 50% [31]. The data on the type of surgery for primary tumors are listed in Table 1. Altogether 16 patients had pT4 tumor and 9 patients had an R2 tumor resection. After surgical procedures macroscopic tumor was present on the trachea in 6 cases, the esophagus in 4 cases, the larynx in 2 cases, the carotid artery in 2 cases, mediastinal vessels in 2 cases, recurrent laryngeal nerve in 1 cases and the prevertebral fascia in 1 case. None of our patients required total laryngectomy or any other radical procedure. Infiltration of trachea was managed surgically by tracheal shaving, and in one case, by tracheostomy.

Metastases in regional lymph nodes were treated surgically by modified radical neck dissection in nine patients; in seven as part of primary treatment and in two because of disease recurrence. Levels 2–6 and levels 2–5 were removed in five and four cases, respectively.

RAI was used for the ablation of thyroid remnant tissue in 87 patients (80%) treated with surgery. The ablation dose was 2.98-3.7 GBq (80–100 mCi) of RAI. After surgery, all patients received L-thyroxine therapy for thyrotropin (TSH) suppression. RAI was used also for the treatment of distant metastases and inoperable locoregional recurrences. Patients with distant metastases underwent total thyroidectomy prior to RAI treatment in order to increase the uptake of iodine in metastases and to speed up treatment. Before 2002, the serum concentration of TSH of >30 mU/L had been achieved by a 4–6-week withdrawal of L-thyroxine suppression therapy. Since 2002, recombinant human TSH (rhTSH)-aided RAI therapy has been used in elderly patients with concomitant diseases, a history of severe hypothyroid or compressive symptoms and/or evidence of tumor progression during thyroid hormone withdrawal [32].

Sixteen of our patients with locoregionally advanced and/or metastatic disease were treated with chemotherapy. Targeted therapy was not used in patients included in the present report. EBRT was done in a total of 27 patients, of whom 23 received EBRT to the neck and superior mediastinum.

### Survival

Disease-specific survival was defined as the period from the first day of primary treatment (surgery, chemotherapy, external irradiation, or radioactive iodine) to death of HCTC or the last follow-up. Overall survival was defined as the period from the first day of primary treatment (surgery, chemotherapy, external irradiation, or radioactive iodine) to death of any cause or the last follow-up.

Disease-free survival was defined as the period from the first day of primary treatment (surgery, chemotherapy, external irradiation, or radioactive iodine) to the radiologic or morphologic diagnosis of recurrence or the last follow-up. Only lymph node metastases were considered as regional recurrence. The median duration of follow-up was 8.75 years (range 0.1-28.1 years).

### Statistical analysis

Univariate analysis was used to identify factors associated with disease-free and disease-specific survival. Disease-specific survival and disease-free interval were compared by log-rank test. All comparisons were two-sided, and a p-value of <0.05 was considered statistically significant. Survival curves were calculated according to the Kaplan–Meier method. Cox’s multivariate regression model was used to identify independent prognostic factors of disease-free and disease-specific survival. For statistical analysis, the statistical package PASW 18 (SPSS Inc., Chicago, IL, USA) was used.

## Results

One hundred eight patients were confirmed as having HCTC at the Institute of Oncology Ljubljana between 1972 and 2011, with a female-to-male ratio of 3.2:1. The median age at diagnosis was 62 years (range 19–87 years).

Tumor diameter ranged from 1 to 18 cm (median 4 cm). Many tumors were locally advanced at presentation. Extrathyroid tumor growth was present in 24 patients (22%) and lymph node metastases were found in 8 patients (7%). Metastatic lymph nodes were found in level 2 in 3 cases, level 3 in 5 cases, level 4 in 4 cases, level 5 in 1 case and level 6 in 2 cases. Distant metastases before therapy were present in 12 patients (11%), and all of these were single-organ metastases. Considering the site of metastatic spread, the lungs were involved in 8 patients, the skeleton in 3 cases, and the kidneys in one patient.

### Disease-free survival

Recurrence was diagnosed in 26 cases: locoregional, distant, and both locoregional and distant in 12, 11, and 3 cases, respectively. The 5-year, 10-year, and 20-year disease-free intervals were 78%, 68%, and 65%, respectively. After surgical therapy of locoregional recurrence, six patients had been without evidence of disease for 0.5-20 years (median 4 years).

Univariate analysis (Table 2) showed that ten factors were associated with disease-free survival (p < 0.05). All factors that showed statistical correlation (p < 0.05 or less) with duration of survival and treatment-related factors were included in the multivariate analysis. Independent prognostic factors for disease-free survival were: gender, age of patients, regional metastases, and residual tumor after surgery. Relative risks of disease-free survival of subgroups are shown in Table 3. Shorter disease-free survival was 2.97 times more likely in men compared to women. Shorter disease-free survival was 17.04 times more likely in patients older than 45 years of age than in younger patients and 7.56 times more likely in patients with regional metastases than in patients without regional metastases. Shorter disease-free survival was 5.05 and 1.24 times more likely in patients with macroscopic and microscopic residual tumor than in patients without residual disease, respectively.

**Table 2:** **Univariate statistical analysis of disease-free survival**

Factor	Subgroup	Without recurrence (N = 70)	With recurrence (N = 26)	p-value
**Gender**	Female	59	14	0.001
	Male	11	12	
**Age (years)**	44 or less	20	1	0.03
	45 or more	50	25	
**Tumor diameter (cm)**	0-4	44	11	0.10
	4.01 and more	26	15	
**pT tumor stage**	pTx, pT1 or pT2	38	5	0.001
	pT3	28	13	
	pT4	4	8	
**N stage**	N0	68	22	0.049
	N1	2	4	
**M stage**	M0	70	26	-
	M1	0	0	
**Tumor differentiation**	Well	48	13	0.06
	Moderate or poor	22	13	
**Vascular invasion**	Minimal or suspected	47	16	0.47
	Extensively	23	10	
**Capsular invasion**	No or minimal	34	16	0.34
	Transcapsular	36	10	
**Thyroid surgical procedure**	Total or near-total thyroidectomy	55	16	0.05
	Lobectomy or less	15	10	
**Residual tumor after surgery**	R0 – without residual tumor	62	15	0.005
	R1 – microscopic residual tumor	5	6	
	R2– macroscopic residual tumor	3	5	
**Neck dissection**	No	70	22	0.005
	Yes	0	4	
**Radioiodine ablation**	No	16	3	0.26
	Yes	54	23	
**Therapy with radioiodine**	No	65	12	0.001
	Yes	5	14	
**External beam radiotherapy**	No	62	17	0.01
	Yes – neck and mediastinum	8	9	
**External beam radiotherapy**	No	64	15	0.001
	Yes – any site	6	11	
**Chemotherapy**	No	66	22	0.11
	Yes	4	4	
**Outcome**	Alive without disease	52	0	-
	Alive with disease	0	14	
	Dead of disease	0	11	
	Dead of other causes	14	1	
	Lost to follow-up	4	0

**Table 3:** **Multivariate analysis of disease-free survival**

Factor	Subgroup	Odds ratio	Odds ratio (95% CI)	p-value
**Gender**	Female	1	1	0.013
	Male	2.97	1.25 – 7.04	
**Age (years)**	44 or less	1	1	0.007
	45 or more	17.04	2.19 – 132.46	
**N stage**	N0	1	1	0.001
	N1	7.56	2.26 – 25.27	
**Residual tumor after surgery**	R0	1	1	0.04
	R1	1.24	0.36 – 4.23	0.726
	R2	5.05	1.66 – 15.38	0.04

The likelihood of malignancy as calculated by logistic regression (model summary: chi-square =44.596; 5 degrees of freedom; P = 0.0001; -2 log likelihood of 177.131).

### Disease-specific survival

Disease-specific survival of our patients ranged from 0.1 to 28 years. The 5-year, 10-year, and 20-year disease-specific survival were 96%, 88%, and 67%, respectively.

By the end of the study, 68 patients were still alive (52 had no evidence of disease, 16 were alive with disease), 5 patients were lost to follow-up, 15 patients died of causes unrelated to primary disease, while 20 patients died of thyroid carcinoma. Of the latter, 18 patients died of distant metastases, and the remaining 2 patients both died of distant and locoregional progression of the disease.

**Figure 1:**
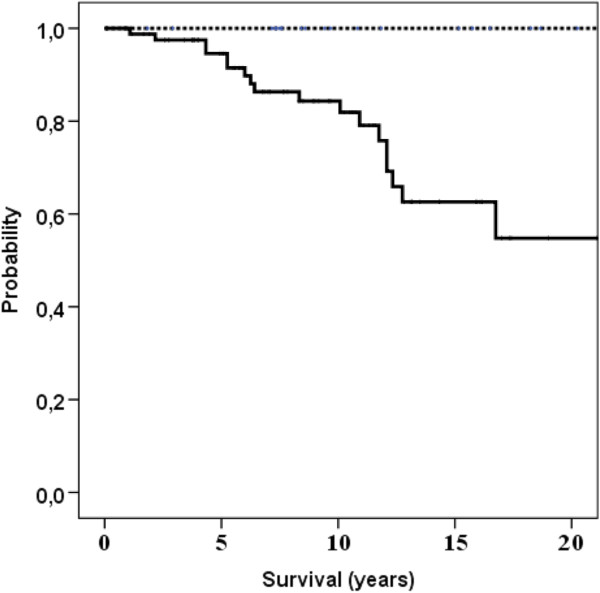
**Age of patients and disease-specific survival.** Spotted line: age 19–44 years. Solid line: 45 – 87 years.

**Figure 2:**
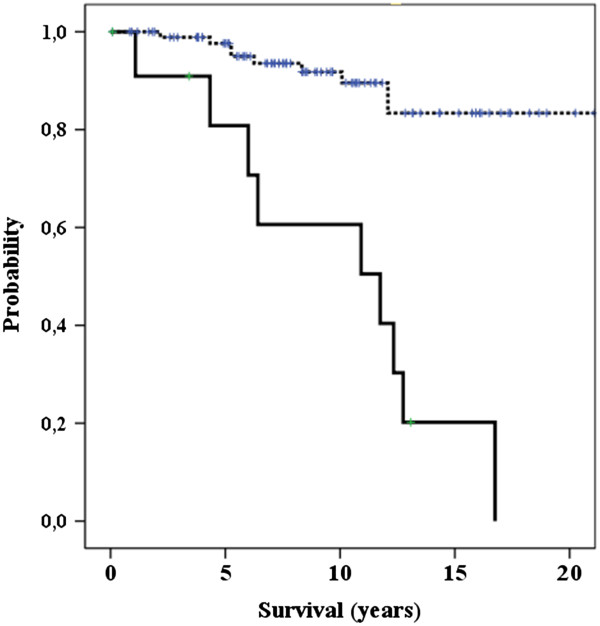
**Distant metastases and disease-specific survival.** Spotted line: without metastases Solid line: with metastases

**Figure 3:**
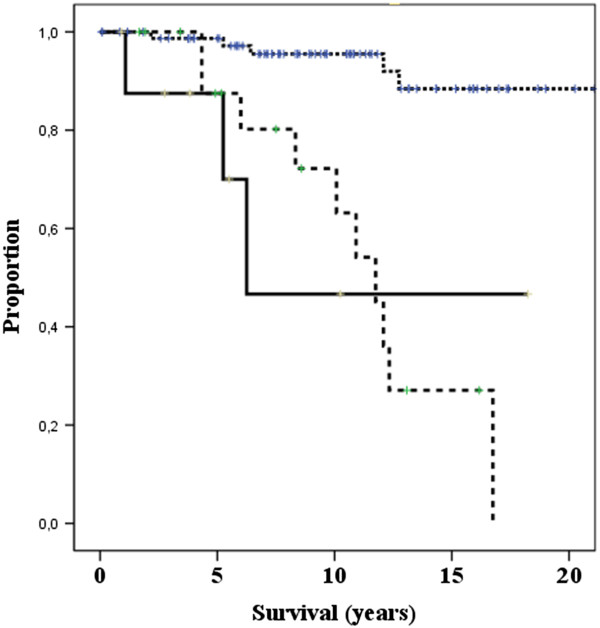
**Residual tumor after surgery and disease-specific survival.** Spotted line: R0 resection. Dotted line: R1 resection. Solid line R2 resection.

Univariate analysis (Table 1) showed that eleven factors were associated with disease-specific survival (p < 0.05). All factors that showed statistically univariate correlation (p < 0.05 or less) with duration of survival and treatment-related factors were included in the multivariate analysis. Independent prognostic factors for disease-specific survival were (p < 0.001): age of patients (Figure 1), distant metastases (Figure 2), and residual tumor after surgery (Figure 3).

The 10-year survival of our patients without distant metastases at presentation was 92%, while in those with distant metastases it was 60%. Patients with initial metastases survived 0.1-16.75 years (median 11.75 years). The survival of eight patients with initial lung metastases was 3.4-16.75 years (median 11.75 years).

## Discussion

There are only a limited number of publications about prognostic factors for disease-specific survival in patients with HCTC from different single institutions [1, 2, 6, 7, 9, 11, 14, 15, 17, 19, 23]. In these publications, 20 different prognostic factors were reported. Prognostic factors were related to: patient’s characteristics [6, 19], tumor [6, 9, 11, 17, 19, 23], extent of disease [1, 6, 9, 17, 19, 23] and treatment [1, 6, 19, 23]. The majority of series included only a very limited number of patients, therefore the authors of these studies could perform only univariate statistical analysis. To our knowledge, so far only six publications [1, 2, 6, 11, 19, 23] have reported the results of multivariate regression analysis of prognostic factors for disease-specific survival in patients with HCTC. The following prognostic factors were reported: age of patients [2, 6], gender [2], marital status [6], tumor size [2, 6, 17], extrathyroidal invasion [2, 6, 19], tumor stage [23], regional metastases [23], distant metastases [6, 19], capsular invasion [11], multifocal disease [19], solid pattern of growth [11], and extent of surgery [23]. In our patients, the independent prognostic factors for disease-specific survival were: age of patients, distant metastases, and residual tumor after surgery. Two of the previous six papers were population-based [2, 6], while two were clinicopathologic [11, 17]. To continue, two papers reported data from major cancer comprehensive centers covering large populations [19, 23]. However, both of them included a smaller number of patients than our study, therefore our paper provides more robust data on a larger number of prognostic factors.

The mean age of our patients at diagnosis was 58 years, which is in accordance with the majority of reports in the literature [1, 6, 10, 14, 15, 23]. Our results confirm the findings in the literature [2, 6, 17] and show that patients’ age is an independent prognostic factor for both disease-specific survival and disease-free interval. In patients younger than 45 years of age, shorter disease-free survival was as many as 17.04 times more likely than in older patients.

Distant metastases were an independent prognostic factor for disease-specific survival in our patients. In patients with distant metastases, the risk of shorter survival was 3.3 times higher than in patients without distant dissemination. This relative risk was 5.3 in the study of Lopez-Penabad et al. [19].

Residual tumor after surgery was another independent prognostic factor for disease-specific survival in our population of patients. Our multivariate analysis also included the following confounding factors related to locoregional tumor extension and treatment: tumor size, tumor stage, regional metastases, capsular invasion, extrathyroidal invasion, and extent of surgery.

The recurrence rate in our patients was 27%, which is comparable to reports in the literature [1, 17, 19, 23]. Khafif et al. reported that locoregional recurrence occurred in only 4 of 42 patients (10.5%) [14]. The probable cause for their low recurrence rate is the obvious difference in patient selection between our and their study groups. Their patients received primarily private consultations with only two surgeons. Therefore, HCTC was usually diagnosed at an earlier stage [14]. On the other hand, our patients had a lower recurrence rate compared to patients from another two tertiary cancer comprehensive centers: Royal Marsden Hospital and Sloan-Kettering Cancer Center, with the recurrence rate of 34% and 43%, respectively [23, 17]. Possibly, the lower recurrence rate in our patients was related to more frequent use of adjuvant multimodal therapy.

The 10-year disease-free survival was 68% in our patients, while it was reported to be 40.5%, 43%, and 61% by Kushcayeva et al. [1], Mills et al. [23], and Stojadinovic et al. [17], respectively. To our knowledge, only six reports [1, 14, 15, 17, 22, 23] were published about disease-free survival and 15 factors were found to correlate with recurrence of HCTC. Predictive factors were related to: patient’s characteristics [1], tumor [14, 15, 17, 22], extent of disease [1, 15, 17, 23] and treatment [1, 23]. However, the majority of previous papers had a small number of patients, and only two publications [15, 23] reported the results of multivariate logistic regression analysis of the disease-free interval in patients with HCTC. Our multivariate analysis showed that independent prognostic factors for disease-free survival were: gender, age of patients, regional metastases, and residual tumor after surgery. Regional metastases have already been a known independent prognostic factor from the study of Stojadinovic et al. [15]. Extrathyroidal invasion was another independent prognostic factor in their study [15]. However, in our study group, extrathyroidal invasion was a prognostic factor by univariate analysis only and was not an independent factor, while residual tumor after surgery was an independent prognostic factor. Furthermore, an old clinical observation that younger patients and females have a favorable prognosis was statistically proven by our results.

The 10-year disease-specific survival in our patients was 88%, while it was 49%, 64%, and 73% in studies reported by Kushcayeva et al. [1], Mills et al. [23], and Stojadinovic et al. [17], respectively. It should be stressed that survival rates represent the result of both selection bias, i.e. more advanced disease in larger tertiary centers, and the effectiveness of different treatment modalities used in patients. Fortunately, SEER [6] and our data show that nowadays the majority of patients with HCTC have a favorable prognosis. Total thyroidectomy and RAI ablation of thyroid remnant tissue enable early detection of tumor recurrence. Furthermore, it is possible that such treatment affects disease-free and disease-specific survival. In addition, our results also show that long-term survival can be obtained also in patients with locoregionally advanced and metastatic disease, if they are treated multidisciplinary. Chemotherapy before surgical procedure may be effective in order to decrease the tumor size in HCTC [31]. Our group reported that RAI therapy may be effective in patients with metastatic HCTC [18] and that recombinant human thyrotropin-aided RAI therapy may be effective in patients with metastatic HCTC [32]. Since residual tumor after thyroid surgery is an independent prognostic factor for disease-specific as well as disease-free survival, an effective locoregional therapy is mandatory. Therefore, EBRT should be used in cases of residual tumor.

The limitation of our study is that the results of treatment and survival are reported only from a single institution. Another limitation is that not all our patients were treated uniformly because during a 40-year time frame, they were treated by many surgeons and oncologists. Our study is observational and not randomized, thus it is not possible to draw conclusions about the impact of treatment on patients' survival. However, the advantage of our study is that it included all patients with HCTC from Slovenia treated in the period from 1972 to 2011. During this period, all patients with thyroid carcinoma were referred to the only tertiary referral center for postoperative treatment and follow-up of patients with thyroid carcinoma in our country. Thus, our data are more comparable to the study reported by Goffredo et al. [6], a population-level analysis of 3,311 patients from the Surveillance, Epidemiology, and End Results (SEER) database from 1988 to 2009 in the USA, than to reports from cancer comprehensive centers. Patients with more advanced disease are more often treated in these centers than in community hospitals [9, 11, 15, 17, 19, 22–24]. Goffredo et al. [6] reported that the female-to-male ratio was 3.22:1, mean age of patients was 58 years, mean tumor size was 3.6 cm, and extrathyroid extension was present in 14% of cases. Initially, only 5% of their patients had regional and 5% distant metastases. However, our patients had more advanced disease: mean tumor size was 5 cm, extrathyroid extension was present in 22% of cases, 7% of patients had regional and 11% distant metastases. Probably, our patients had more advanced disease because Slovenia is an endemic goiter region [33]. However, our patients had similar 10-year disease-specific survival compared to the SEER database patients reported by Goffredo et al. [6]. This can be explained by more effective treatment of our patients. Total thyroidectomy enables more effective adjuvant treatment with different modalities (radioactive iodine, EBRT and/or chemotherapy) resulting in longer survival, which is particularly important in patients with more advanced HCTC. Total thyroidectomy, RAI ablation of the thyroid remnant, and EBRT were done in Goffredo’s [6] and our patients in 75%, 45% and 4%, and 71%, 87% and 27%, respectively.

## Conclusion

Independent prognostic factors for disease-specific survival were: age of patients, distant metastases, and residual tumor after surgery. Independent prognostic factors for disease-free survival were: gender, age of patients, regional metastases, and residual tumor after surgery.

### Consent

Written informed consent was obtained from the patient for the publication of this report and any accompanying images.
